# One-Year Outcomes of Modified Technique for Scleral Fixation of a Three-Piece Intraocular Lens Without Conjunctival Opening

**DOI:** 10.3389/fmed.2022.856800

**Published:** 2022-06-02

**Authors:** Hongfei Ye, Shiguang Zhang, Wei Mi, Ping Fei, Peiquan Zhao

**Affiliations:** ^1^Department of Ophthalmology, Xinhua Hospital Affiliated to Shanghai Jiao Tong University School of Medicine, Shanghai, China; ^2^Department of Ophthalmology, Zhenjiang Ruikang Hospital, Zhenjiang, China

**Keywords:** intraocular lens, three-piece, scleral fixation, Hoffman pockets, outcomes, modified technique

## Abstract

**Purpose:**

This study aimed to present the 1-year follow-up of a modified technique for scleral fixation of three-piece intraocular lens (IOLs) without conjunctival incision.

**Materials and Methods:**

A retrospective chart review of a consecutive series of 10 eyes of nine patients who underwent scleral IOL fixation using the modified technique was performed. Data were collected 1 year after surgery for all patients.

**Results:**

The range of follow-up time was between 1 year and 31 months. At the last follow-up point, the IOL was well-positioned and the visual acuity was good (as limited by primary diseases). Short-term complications included pupillary IOL capture (*n* = 1) and decreased intraocular pressure (*n* = 1), and no long-term complications were observed.

**Conclusion:**

Outcome data support this technique as a viable option for the management of secondary IOL fixation with flexible usage of more designs of IOLs.

## Introduction

Scleral fixation of posterior chamber intraocular lens (PCIOLs) is a widely accepted way to restore aphakic eyes without enough capsule, such as congenital and acquired zonular weakness, posttraumatic subluxation, and post lensectomy. Although several sutureless scleral fixation methods have been reported in recent years, such as the flanged haptic technique (1) and the scleral tunnel approach (2, 3), the scleral suture fixation of PCIOL is still one of the most effective methods and offers relatively easy management of potential surgical complications (4).

CZ70BD IOL (Alcon Laboratories, Inc., Fort Worth, United States) is a commonly used scleral-sutured IOL that is made of polymethyl methacrylate (PMMA) and has an optic diameter of 7 mm. While such a sizable rigid IOL requires a large incision and risks intraoperative damage and postoperative complications (5), the use of foldable IOL refines this surgery into a small incision; however, most of the IOLs reported for scleral fixation in previous studies were specially designed with closed-loop haptics, such as Akreos AO IOL (Bausch & Lomb, Inc., Rochester, United States) (6, 7) with four eyelet haptics and C-flex IOL (Rayner Intraocular Lenses Ltd., East Sussex, United Kingdom) with two closed-loop haptics (8). The options of foldable IOLs for scleral fixation are still limited. Therefore, developing novel fixation techniques for flexible usage of various designs of IOLs represents a general trend and fits in with different conditions.

In this study, we propose a modified technique for the scleral fixation of a secondary foldable three-piece PCIOL, which demonstrates surgical success in ten eyes. The long-term outcomes are presented to evaluate the reliability and reproducibility of this novel technique.

## Subjects and Methods

Institutional Review Board approval and Ethical Review Board approval were obtained from Xinhua Hospital Affiliated to Shanghai Jiao Tong University School of Medicine. The study adhered to the principles of the Declaration of Helsinki. A retrospective chart review consisting of 10 eyes of nine patients who underwent modified transscleral suture fixation of a 3-piece pre-loaded PIOL (PY60AD, HOYA Medicals, Tokyo, Japan) between December 2018 and July 2020 was performed. Patients were followed up for at least 1 year after surgery.

Data collected included surgical indications, primary surgery (if applicable), the time interval between the present and primary surgery, and relevant ocular and systemic history. Complete ophthalmic examination was conducted for all the patients, including best-corrected visual acuity (BCVA), intraocular pressure (IOP), refraction, slit-lamp biomicroscopy, dilated fundus examination, axial length, and corneal endothelial density (ECD) preoperatively and at 1 month and the last visit postoperatively. The presence of postoperative complications was also recorded. IOL tilt and decentration were measured and calculated automatically with swept-source anterior segment optical coherence tomography (SS-ASOCT, CASIA2; Tomey Corp., Nagoya, Japan) under the mode of a 3-dimensional (3D) scan at the last visit under mydriatic conditions using a mixture of 0.5% tropicamide and 0.5% phenylephrine hydrochloride (Mydrin-P, Santen Pharmaceutical, Osaka, Japan) according to the method previously described (9, 10). Briefly, CASIA2 uses a swept-source laser with a 1,310-nm wavelength at a frequency of 0.3 s and provides higher resolution images of IOLs. Using the IOL scan mode, 8 distinct ASOCT images from 8 different angles (namely 0–180, 90–270, 23–203, 113–293, 45–225, 135–315, 68–248, and 158–338) are obtained and a 3D image is created (9). IOL tilt and decentration are directly generated by the built-in software (Version SS2000) relative to the visual line, and the detailed extent and azimuth (the orientation of IOL tilt and decentration in degree) are present beside the image.

### Surgical Technique

**FIGURE 1:**
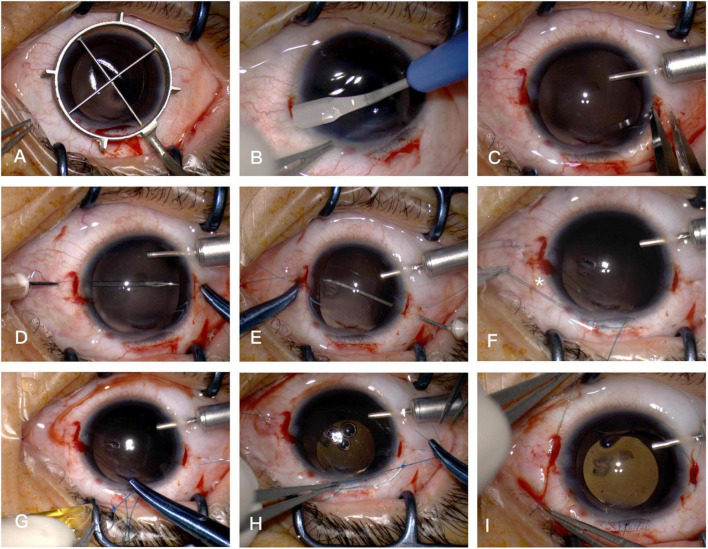
Surgical steps. **(A)** Two location imprints at 3 o’clock and 9 o’clock are made by an ophthalmic marker. **(B)** Two scleral pockets are dissected by a crescent blade posteriorly from the limbus, achieving a thickness of 300 μm and a length of 3.0 mm. **(C)** After anterior chamber infusion, puncture points are marked at the middle line of the pocket beds 2.0–2.5 mm posterior to the limbus on both sides. **(D)** The straight needle of a double-armed 9-0 polypropylene suture (with two curved needles) is introduced at one puncture point at 9 o’clock through the conjunctiva and full thickness of the Hoffman pocket, passing through the anterior chamber to the opposite side, then docked into the opening of a 27-G needle, which is introduced at the other puncture point at 3 o’clock, then removed externally. **(E)** The straight needle is again passed backward through the conjunctiva and the full thickness of the scleral pocket 1.0–2.0 mm adjacent to the first pass of the needle, threading through the anterior chamber and docked again with the 27-G needle, and guided out in the same way 1.0–2.0 mm adjacent to the puncture point at 9 o’clock. **(F)** The pairs of sutures are retrieved externally through the superior incision and cut into two halves. The star symbol demonstrates one end of the double sutures to tie on the leading haptic. **(G)** The leading haptic of the pre-loaded PIOL is pushed out of the cartridge and tied with one of the double sutures at the junction of the enlarged haptic end. **(H)** After the leading haptic and the folded IOL optic is injected into the anterior chamber, the trailing haptic left externally is tied with the other double sutures. **(I)** The two pairs of suture ends are retrieved through the scleral pocket opening pulled out by the Sinskey hook and knotted *via* tension adjustable knot to center the optic of the IOL.

The step-by-step surgical procedure was demonstrated in Figure 1. After the external incision of the superior scleral tunnel is made 2 mm posterior to the limbus at 11 o’clock, an ophthalmic marker is placed and pressed on the cornea, making two location imprints at 3 o’clock and 9 o’clock. Using a crescent blade, two partial-thickness grooves (300 μm-depth, 3.0 mm-long) are made on the corneal limbus at the two imprints, followed by dissection of two scleral pockets posteriorly from these limbal grooves. The pockets are extended perpendicular to the limbus and continued for 3.0 mm, maintaining a uniform depth in the sclera. Then, a paracentesis is made at 8 o’clock by a 15-degree lance tip blade for 20-G infusion, and another paracentesis is made using a 3.0 mm sharp-tip keratome to complete the superior sclerocorneal incision.

Afterward, a double-armed 9-0 polypropylene suture (Mani Inc., Tochigi, Japan) with two straight needles (or one straight needle and one curved needle) is used for IOL fixation. Two puncture points are marked by calipers at the middle line of the pocket beds 2.0–2.5 mm posterior to the limbus on both sides. The straight needle is introduced at one puncture point at 9 o’clock through the conjunctiva and full thickness of the Hoffman pocket, passing through the anterior chamber to the opposite side, then docked into the opening of a 27-G needle, which is introduced at the other puncture point at 3 o’clock and then removed externally. Afterward, the straight needle is again passed backward through the conjunctiva and the full thickness of the scleral pocket 1.0–2.0 mm adjacent to the first pass of the needle, threading through the anterior chamber and docked again with the 27-G needle, and guided out in the same way 1.0–2.0 mm adjacent to the puncture point at 9 o’clock. When visualized in the pupillary area, the pairs of sutures are retrieved externally through the superior incision by a Sinskey hook (or forceps) and cut into two halves, leaving two double-sutured ends for IOL fixation.

Next, the leading haptic of the preloaded PIOL is pushed out of the cartridge and tied with one end of the double sutures at the junction of the enlarged haptic end. After the leading haptic and the folded IOL optic are injected into the anterior chamber, the trailing haptic left externally is tied with the other end double sutures and then pushed subsequently into the eye by the Sinskey hook. By removing the two needles linked to the suture ends passed at 3 o’clock and cutting the suture-loop at 9 o’clock into two single suture ends, each suture end is retrieved through the scleral pocket opening by placing the Sinskey hook into the pocket and pulling and externalizing the trailing suture end. After the PCIOL is placed at the exact position behind the iris, the pairs of sutures at each pocket are knotted *via* a tension adjustable knot to center the optic of the IOL. Each knot is tied into the scleral pocket, and the suture ends were laid flat into each pocket. Finally, the superior incision is sutured, and the 20-G infusion tip is removed with this incision watertight.

The video demonstrates the procedures (Supplementary Video 1). All surgeries were performed by one experienced surgeon (PZ) under general anesthesia for patients under 13 years old or retrobulbar anesthesia for the others. For cases with lens subluxation, lensectomy and complete pars plana vitrectomy (PPV) were performed prior to IOL fixation.

## Results

Ten eyes of nine patients who underwent the procedure were enrolled. All the eyes met the criteria for scleral fixation of PIOL with obviously better preoperative BCVA. The mean patient age was 23.4 ± 22.89 years at the time of the surgery. Four patients had subluxation lens because of Marfan Syndrome (MFS) and received lensectomy and complete PPV before IOL fixation (5 eyes, 50%). The lens in four patients (4 eyes, 40%) were extracted in previous surgeries. One patient (1 eye, 10%) suffered aphakia secondary to ocular rupture. The average axial length was 24.43 ± 1.05 mm. The mean follow-up time was 19.6 ± 7.04 months. Detailed individual patient data are provided in Table 1.

**TABLE 1:** Individual patient characteristics.

Case	Age, years	Gender	Laterality	Axial length, mm	Indication for surgery	Ophthalmolmic comorbidities	Follow-up time, months
1	4	Female	Right	25.33	Crystalline lens subluxation	Marfan syndrome	12
2	5	Male	Right	24.80	Crystalline lens subluxation	Marfan syndrome	21
3			Left	24.68			12
4	7	Male	Left	23.55	Crystalline lens subluxation	Marfan syndrome	24
5	10	Male	Right	23.20	Crystalline lens subluxation	Marfan syndrome	31
6	12	Female	Right	25.98	Traumatic aphakia	Ocular rupture	28
7	27	Male	Left	24.86	Postoperative aphakia	Penetrating injury	21
8	48	Female	Left	25.02	Postoperative aphakia	Vitreous hemorrhage	22
9	52	Male	Left	22.52	Postoperative aphakia	Traumatic RD	12
10	64	Male	Left	24.35	Postoperative aphakia	Traumatic RD and CD	13

*RD, retinal detachment; CD, choroidal detachment.*

As Table 2 demonstrates, the patients’ BCVA ranged from counting fingers to 20/25 preoperatively and 20/125 to 20/20 at the last postoperative follow-up time. The IOP and ECD remained in the normal range before and after surgery. Throughout the follow-up period, one child patient with MFS experienced pupillary capture of IOL optic on the first day after surgery, which was repositioned after pupillary dilation and supine positioning. Another child patient suffered decreased IOP caused by suspected incision leakage on the first day after surgery and was successfully treated with pressure bandaging for 2 days. No other intraoperative or postoperative complications were recorded. The IOL was well-positioned at the last follow-up time (see Table 3).

**TABLE 2:** Comparison of clinical data before and after surgery.

Case	BCVA (Snellen)		IOP, mmHg		ECD, cells/mm^3^		Postoperative complications
			
	Pre	Post	Pre	Post	Pre	Post	
1	20/100	20/80	13	10	3275	3045	None
2	Uncooperative	20/67	Tn	13	Uncooperative	3201	Decreased IOP
3	Uncooperative	20/100	Tn	14	Uncooperative	2996	Pupillary IOL capture
4	20/50	20/40	18	17	3509	3385	None
5	CF/30 cm	20/100	15.6	18.2	3086	3056	None
6	20/25	20/20	13	14	3322	3124	None
7	20/200	20/125	13.2	14.2	1919	1890	None
8	20/25	20/20	23	18.2	2646	2632	None
9	20/133	20/100	13	10.1	2703	2700	None
10	20/67	20/50	15	14.6	1783	1659	None

*BCVA, best-corrected visual acuity; CF, counting fingers; IOP, intraocular pressure; ECD, endothelial cell density; IOL, intraocular lens.*

**TABLE 3:** Parameters of intraocular lens position at the last follow-up time.

Case	Tilt, degree	Azimuth, degree	Decentration, mm	Azimuth, degree
1	1.8	312	0.10	28
2	2.4	320	0.18	105
3	2.8	304	0.14	118
4	3.5	352	0.11	127
5	2.3	308	0.08	24
6	5.0	342	0.14	52
7	6.3	326	0.19	46
8	1.4	338	0.04	182
9	6.8	314	0.17	42
10	7.7	51	0.20	16

## Discussion

Scleral suture fixation of PCIOL is the most widely accepted method and plays an important role in the visual restoration of eyes with inadequate capsule support. In our present study, we for the first time described a modified approach of the scleral Hoffman pocket fixation technique (11), combined with HOYA PCIOL. The clinical observation for at least 1 year demonstrated improved BCVA and a low rate of complication. No patient presented suture knot exposure, suture breakage, or severe IOL tilting or decentration. There were no severe intraoperative or postoperative adverse events during the follow-up period.

The application of the Hoffman pocket technique provides several advantages. Dissection of this pocket starts from a clear corneal incision, avoiding the need for scleral cautery and preserving the integrity of the conjunctiva. This surgical procedure creates a larger surface area for suture passes than traditional triangular scleral flaps (12), allowing the needles to exit inside the dissected pocket as long as they are at a proper distance from the limbus (13). Most remarkably, burying the suture knot in the pocket prevents erosion of the overlying conjunctiva with the potential risk for endophthalmitis and avoids suture breakage and the ensuing IOL malposition or dislocation. In addition, using this technique, the conjunctiva can be preserved at maximum, which is particularly desirable for the patients who previously received or who needed to receive glaucoma infiltration surgery (14). Postoperative complications, such as surgical induced astigmatism (SIA) and corneal edema, are also less likely to happen, and the patients’ comforts are largely improved.

Scleral fixation with the Hoffman pocket technique has been applied for various types of IOLs, while most of the IOLs are used with closed-loop haptics or designed eyelets on the haptics, through which sutures can easily be passed for IOL fixation (15–17). On the other hand, seldom had the 3-piece IOLs been reported to be fixated with this technique, except that Domingues et al. (18) described a cupid technique to perforate and knot at the body of the 3-piece subluxated IOL with 10-0 polypropylene suture. In this study, we for the first-time used Hoffman’s technique in the fixation of a 3-piece pre-loaded IOL, which provides several significant improvements: first, the pre-loaded foldable design of the IOL allows the implantation through a smaller incision, associating with better intraoperative IOP control and less postoperative SIA. Next, the haptics of this IOL, namely HOYA PY60AD, are made of PMMA. The rigid material as well as the C-loop posteriorly angulated thin configuration of its two haptics largely minimize iris chafing, reducing the risk for uveitis-glaucoma-hyphema (UGH) syndrome, pigment dispersion syndrome, and increased IOP. Most distinctively, the end of haptics is designed as an enlarged cone shape, which enables the suture to fixate directly on the haptics by knotting at the junction of haptic ends and at the same time avoid suture slippage (19). Our method provides an effective, simple, safe, and a minimally invasive way for scleral suture fixation with more IOL options.

Concerning complications, pupillary IOL capture was observed in one patient with MFS 1 day postoperatively, and the IOL was reposited followed by pilocarpine therapy without repeated captures. The pupillary capture rate was reported much higher in the pseudophakic eyes of patients with MFS (20–22) because the myopathy of pupil constrictors and dilators in this population gives rise to pliable iris and reverse pupillary block (23, 24). To deal with this situation, preventative intraoperative surgical iridectomy (preferably with small gauge vitrectomy) or postoperative laser iridectomy are recommended (25). We also modified the technique, retreating the fixation plane from 2.0 to 2.5 mm posterior to the limbus for patients with MFS, which noticeably decreased the rate of pupillary IOL capture (not mentioned in this study). Another child patient also suffered from decreased IOP 1 day postoperatively. The Seidel test was done to exclude leakage of the incision, and the IOP returned to normal after pressure bandaging for 2 days. Significantly, the tilt azimuth quite differed in Case 10 compared with other cases, as demonstrated in Table 3. This patient suffered from ocular rupture, resulting in superior temporal traumatic aniridia. The inadequate anterior support of the iris might be the reason for his unique azimuth.

## Conclusion

This surgical technique offers an alternative approach to the management of secondary IOL fixation with flexible usage of more designs of IOLs for patients with insufficient capsular support, achieving a reliable and reproducible procedure with improved anatomical and visual outcomes, reduced complications, and decreased surgical times. However, the limitations of this study include its small sample size and relatively short follow-up period. Future studies with longer follow-up observation are needed to determine its standing among other documented techniques.

## Data Availability Statement

The raw data supporting the conclusions of this article will be made available by the authors, without undue reservation.

## Ethics Statement

The studies involving human participants were reviewed and approved by the Institutional Review Board approval and Ethical Review Board approval were obtained from Xinhua Hospital Affiliated to Shanghai Jiao Tong University School of Medicine. Written informed consent to participate in this study was provided by the participants’ legal guardian/next of kin.

## Author Contributions

HY conceived and designed the analysis and wrote the manuscript. HY, SZ, and WM collected the data and performed the analysis. HY, SZ, PF, and PZ contributed to data collection. PZ carried out final editing and approval. All authors agreed to be accountable for the content of the work.

## Conflict of Interest

The authors declare that the research was conducted in the absence of any commercial or financial relationships that could be construed as a potential conflict of interest.

## Publisher’s Note

All claims expressed in this article are solely those of the authors and do not necessarily represent those of their affiliated organizations, or those of the publisher, the editors and the reviewers. Any product that may be evaluated in this article, or claim that may be made by its manufacturer, is not guaranteed or endorsed by the publisher.

## Supplementary Material

Supplementary Video 1 The surgical procedures of the technique.
